# Emotional neglect and parents’ adverse childhood events

**DOI:** 10.1192/j.eurpsy.2023.2420

**Published:** 2023-06-09

**Authors:** Laura Ylitervo, Juha Veijola, Anu-Helmi Halt

**Affiliations:** 1 Research Unit of Clinical Medicine, Department of Psychiatry, University of Oulu, Oulu, Finland; 2 Medical Research Centre Oulu, Oulu University Hospital and University of Oulu, Oulu, Finland; 3 Department of Psychiatry, Oulu University Hospital, Oulu, Finland

**Keywords:** Adverse childhood events, emotional neglect, transgenerational trauma

## Abstract

**Introduction:**

Emotional neglect means that the child’s emotional and developmental needs are not fulfilled by the parents or other caregivers. Adverse childhood events (ACEs) are a risk factor for mental health problems and impaired parenting skills. The objective here was to examine whether parents’ ACEs increase the child’s risk of experiencing emotional neglect.

**Methods:**

The participants in the present study were members of the Northern Finland Birth Cohort 1986 (NFBC1986). Emotional neglect experiences were measured in 190 members of this cohort by means of the Trauma and Distress Scale (TADS), and ACEs in both parents were measured with a specific questionnaire. A linear regression model was used to examine the association between parents’ ACEs and the children’s emotional neglect scores.

**Results:**

The children’s mean emotional neglect score was 8.11 on a scale from 5 to 25. There was no significant difference between males (mean 8.01) and females (mean 8.19). Only father’s ACEs were associated with child’s emotional neglect score. In the linear regression model, the children’s emotional neglect scores increased by 0.3 points for father’s ACE.

**Conclusions:**

Our findings suggest that father’s ACEs may increase the child’s risk of experiencing emotional neglect. It seems that childhood adversities are transferred from parents to children, but larger samples would be needed to confirm these findings.

## Introduction

Emotional neglect means that the child’s emotional and developmental needs are continuously ignored by the parents, so that the experiencing of emotional neglect may be said to be associated with the type of the relationship between the child and his or her caregivers. Probably, one of the most widely recognized models for these relationships is Bowlby’s attachment theory, first introduced in 1958 [1]. According to this theory, the type of child–parent relationship can be determined by examining how the child reacts in stressful situations with respect to the caregiver. This again depends on how the child views the caregivers: that is, whether they are able to provide the nurture and support that the child needs. Emotional neglect comes into the picture when the caregiver cannot fulfill the child’s basic emotional needs, because of impaired parenting skills and responsiveness, possibly due to mental health problems [2] or substance use [3], for example. Emotional neglect may lead to an insecure or anxious attachment style, which in turn could increase the risk of anxiety and other mental health problems [4].

Emotional neglect is one type of adverse childhood experiences (ACEs), but there is a lack of research on its prevalence in general population. For example, Stoltenborgh et al. (2013) concluded a meta-analysis on emotional neglect, and they pointed out that the 13 suitable studies they found about emotional neglect is very little compared to other ACE domains, such as sexual abuse [5]. Mathews et al. (2020) found 23 studies in their meta-analysis, which still falls short in comparison to other ACEs [6]. There is no consistent and established questionnaire to measure emotional neglect, which leads to difficulties comparing prevalence numbers across studies.

It seems that childhood adversities are transferred from parents to children at least in some form. ACEs may influence mental health even at a young age, but also in adulthood [7–9]. Emotional and physical neglect and abuse experienced in the childhood are risk factors for depression and anxiety [10, 11] and for lifelong alcohol dependence [12]. Taken together, if parents have experienced ACEs, they will have a higher risk of mental health problems, and since having a depressed parent, for example, is considered an adverse childhood event, the child of such a parent will be at risk, leading to a vicious cycle in which adversities can be passed on for generations.

Another example of how this cycle may be initiated is post-traumatic stress disorder (PTSD). Muzik et al. found that mothers with PTSD have a higher risk for impaired bonding with their children [13]. According to previous literature, parents with PTSD are more prone to aggressive or hostile behavior toward their children [14, 15], are emotionally less available [15], perceive their child more negatively [15] and are less satisfied with their parenting [14, 15]. Children learn emotional regulation from their parents, and parents’ PTSD has been found to be associated with child’s internalizing and externalizing behavior as well as emotional regulation [16, 17]. On the other hand, Greene et al. found that if a parent with PTSD is capable of responsive parenting behavior, the adverse effects could be avoided [18].

In addition to other psychiatric conditions, substance use disorders can potentially lead to emotional neglect. Meulewaeter et al. [3] studied mothers with substance use disorders and how they reflected their own parenting experiences and bonding with their children. It became apparent from the interviews that these mothers had noticed how their substance use had impacted their parenting skills and how they had emotionally neglected their children. They also described having had similar experiences in their own childhood concerning their relations with their mothers.

This brings us back to the cycle of adverse childhood events and the phenomenon of the transfer of traumatic experiences across generations. Leifer et al. [19] conducted an intergenerational study to explore transmission of the risk of sexual abuse. Their material included three generations, children and their mothers and grandmothers, and they found that the mothers of children who had experienced sexual abuse themselves had a history of childhood abuse and neglect and had more often had conflicting relationships with their own mothers [19]. Taken together, these previous research findings suggest that if the first-generation mother is traumatized or otherwise has impaired parenting skills, this could cause mental health problems for her children, and furthermore, that if the second-generation parents have mental health problems, then the third generation could also be at risk, and so on.

We examined here a sample of 190 cohort members and their parents to see whether the parents’ ACEs increased the risk of their children experiencing emotional neglect. We hypothesized that the more ACEs the parents had experienced, the more emotional neglect their children would experience.

## Methods

### Study setting

#### The NFBC1986 material

This study is based on the Northern Finland Birth Cohort 1986 (NFBC1986), which is a prospective longitudinal research program covering 99% of all births in Northern Finland with their calculated term between July 1, 1985, and June 30, 1986 (*n* = 9,479, live born 9,432) [20]. Data on the participants have been collected from health registers, questionnaires, and clinical examinations for a period extending from prenatal care to the present day. The NFBC1986 data give a representative sample of the general population in Finland and represent one of the largest birth cohort studies with high genetic and ethnic homogeneity. The design and population of the NFBC1986 are described in full in Järvelin et al. [21].

#### Population

Out of the 1,396 members of the NFBC1986 cohort invited to participate in this study, 471 (34%) accepted and answered the Trauma and Distress Scale (TADS) questionnaire at the age of around 26 years [22]. These participants will for clarity be referred to below as “children” even though they were adults at the time of answering the TADS questionnaire.

**Figure 1.**
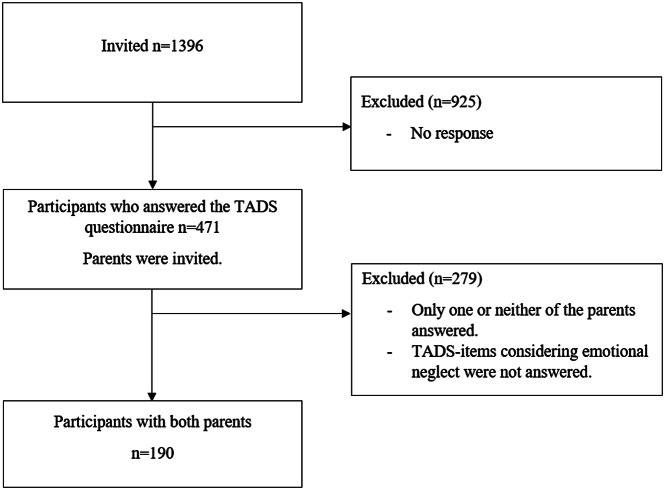
Participant selection.

The participants’ parents were also invited to join the study and to fill in a questionnaire concerning their adverse childhood experiences (ACEs). We then excluded those participants for whom both parents refused to answer the ACEs questionnaire (*n* = 279, 59%) as well as those who had themselves not answered the TADS questions concerning emotional neglect (*n* = 2, 0.4%). This gave a total series of 190 children and their parents (i.e., 190 mothers and 190 fathers). Participant selection is illustrated in Figure 1.

The participants gave their consent for their parents to be contacted and for their own data to be linked to their parents’ data. Conversely, all the parents consented to their data being linked to their child’s data and to the data of the other biological parent. The protocol was approved by the Ethical Committee of the Northern Ostrobothnia Hospital District.

### Instruments

#### Trauma and Distress Scale

The Trauma and Distress Scale (TADS) questionnaire that was used here to measure childhood adversities is a self-reported instrument developed by Patterson et al. [23] and translated into Finnish by Salokangas et al. [24]. It consists of 43 items and divides childhood adversities into five domains: emotional neglect, emotional abuse, physical neglect, physical abuse, and sexual abuse.

We focused on the five questions concerning emotional neglect: 5) When I was young, I felt valued or important, 8) My family were emotionally warm and loving, 13) When I was young, my family looked after each other, 21) I respect myself, and 40) My family was supportive and encouraging when I was young. These questions were answered on a scale from 1 to 5, where 1 denoted “never” and 5 “almost always.”

Because of the phrasing of the other questions in TADS, the points from the questions concerning emotional neglect had to be reversed. Thus, the more points a subject scores on these five questions (range 5–25), the more emotional neglect that person experienced in childhood.

#### Questionnaire for the parents

The parents of the NFBC1986 members were invited to answer a questionnaire concerning their childhood, personality, and substance use that formed part of the Health 2000 study [25]. Only the questions that outlined the parents’ childhood and relationship with their own parents (i.e., the grandparents of the NFBC1986 members) were considered here. The questionnaire included 13 items related to adverse childhood events, such as being seriously ill, bullied, or assaulted, having a parent with mental illness or problems with alcohol, or divorce of the parents. These questions were answered on a scale from 1 to 3, where 1 denoted “no,” 2 “cannot say,” and 3 “yes.” Only the “yes” answers were accepted for calculating the number of ACEs for each parent. The original questionnaire is available in Supplementary Material.

#### Confounders

The age and education of the parents and the child’s gender were accepted as confounders in the statistical analysis. The parents’ age was their age at the time when their child was born, as calculated from their dates of birth. Education of the parents and the children was assessed in terms of four levels: 1 = 9 years of school and/or vocational training, 2 = general or vocational upper secondary school, 3 = university of applied sciences, and 4 = university.

### Statistical analysis

The children’s emotional neglect scores and the numbers of parents’ ACEs were analyzed to investigate the possible association between emotional neglect and parents’ adverse childhood events. The statistical analysis was conducted with IBM SPSS Statistics. Assumption of independence of all variables was tested and met.

Due to skewed distribution of child’s emotional neglect score, Spearman’s correlation coefficients were used to see which variables were associated with the emotional neglect score. We also used Kruskal–Wallis test to see if the amount of parents’ ACEs had a different effect on child’s emotional neglect score. For this test, parents’ ACEs were divided into four classes: 0 = no ACEs, I = one ACE, II = two or three ACEs, and III = four or more ACEs (a similar categorization to that used by Kananen et al. [26]).

For linear regression model, assumptions of linear association, heteroscedasticity, no multicollinearity and normality of residuals were tested and met. Normality of residuals was tested with Normal Probability Plot.

## Results

Basic demographic information concerning the children and their parents is shown in Supplementary Tables 1 and 2. Supplementary Table 1 also shows results of the drop-out analysis, which revealed that the mean emotional neglect score of the excluded participants (*n* = 279) was higher that included participant’s emotional neglect score (9.23 and 8.11, *p* = 0.001). Other variables did not significantly differ between the included and excluded participants (see Supplementary Table 1).

The numbers of adverse childhood events reported by the parents are outlined in the Table 1 in four ACE groups. According to these categories, 8.9% of mothers and 5.3% of fathers had four or more ACEs (see Table 1).

**Table 1.** Parents’ ACEs divided into four classes

	Mothers *N* (%)	Fathers
No ACEs	85 (44.7%)	78 (41.1%)
1 ACE	44 (23.2%)	56 (29.5%)
2–3 ACEs	44 (23.2%)	46 (24.2%)
4 or more ACEs	17 (8.9%)	10 (5.3%)

Abbreviation: ACEs, adverse childhood events.

**Figure 2.**
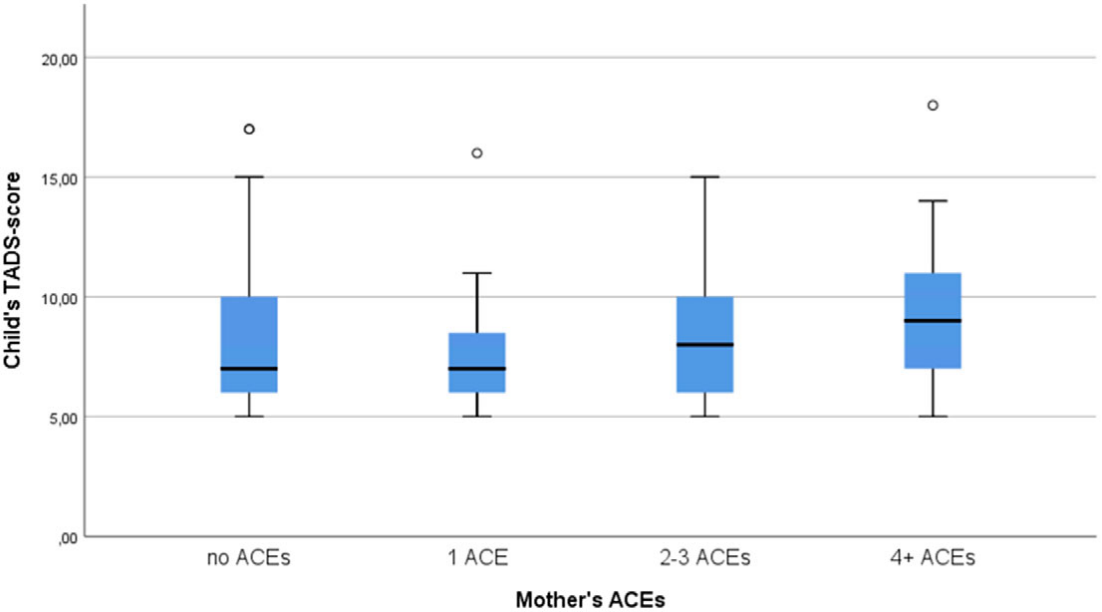
Box-plot figure of the association between mother’s ACEs and child’s emotional neglect score.

**Figure 3.**
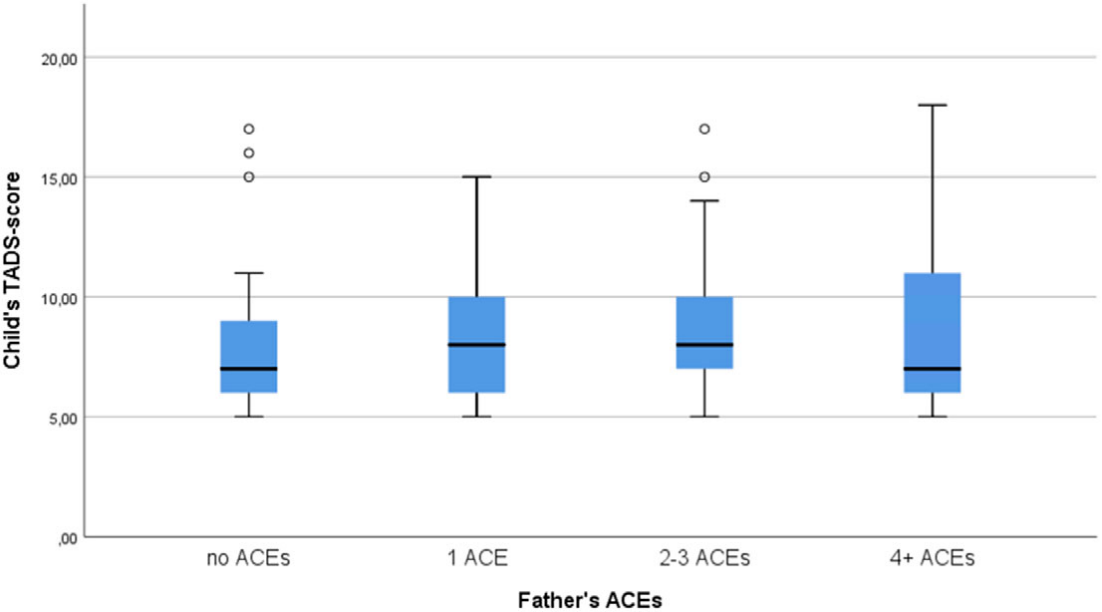
Box-plot figure of the association between father’s ACEs and child’s emotional neglect score.

We examined children’s emotional neglect scores in these four groups and found an ascending trend (see Figures 2 and 3). The Kruskal–Wallis test found no significant difference between the four classes (*p* = 0.089 for father and *p* = 0.109 for mother).

We also tested if any of the parents’ individual ACE domains were associated with the child’s emotional neglect score. For mothers, experience of financial challenges of the family (*p* = 0.008) and divorced parents (*p* = 0.009) were associated with child’s emotional neglect score. For fathers, experience of mother’s alcohol abuse (*p* = 0.011) was associated with the child’s emotional neglect score.

Table 2 shows the correlations between child’s emotional neglect score, parents’ ACEs, and the confounders. Spearman’s correlation analysis showed a minimally weak correlation between the number of father’s ACEs and child’s emotional neglect score (*r* = 0.150, *p* = 0.039). Mother’s ACEs were not associated with the child’s emotional neglect score (*p* = 0.406). Father’s education had a negative correlation to child’s emotional neglect score (*r* = −0.176, *p* = 0.017; see Table 2).

**Table 2.** Correlations between child’s TADS-score, parents’ ACEs, and confounders

	Spearman’s correlation coefficient	Significance (*p*-value)
Mother’s ACEs	0.061	0.406
Mother’s age	0.073	0.320
Mother’s education level	−0.093	0.213
Father’s ACEs	0.150	**0.039**
Father’s age	0.074	0.313
Father’s education level	−0.176	**0.017**
Child’s gender*	0.021	0.772

Abbreviations: ACEs, adverse childhood events; TADS, Trauma and Distress Scale.

* 1 = boy, 2 = girl.

Bolded = statistically significant *p*-value

As only father’s ACEs and education level were associated with the child’s emotional neglect score, the linear regression model was conducted only for father. The results of the linear regression model are shown in Table 3. The model is a multilinear analysis with father’s education and age as confounders. This regression model points to a statistically significant correlation between the father’s ACEs and the child’s emotional neglect scores (*p* = 0.045). In this model, the confounders were not statistically significant. According to the model, child’s predicted emotional neglect score is 6.3 + 0.343 (father’s ACEs) points; in other words, the child’s emotional neglect score increased by 0.343 points for each father’s ACE (see Table 3).

**Table 3.** Linear regression between father’s ACEs and child’s emotional neglect score

	Unstandardized coefficients beta	Standardized coefficients beta	95% confidence interval	*p*-Value
Constant	6.303		3.473–9.133	<0.001
Father’s ACEs	0.343	0.152	0.007–0.679	0.045
Father’s education	−0.356	−0.102	−0.864–0.152	0.169
Father’s age	0.068	0.131	−0.009–0.145	0.085

Abbreviations: ACEs, adverse childhood events

*Note*: Adj. *R* ^2^ 0.028.

## Discussion

Our findings suggest a correlation between the number of father’s ACEs and the child’s emotional neglect score, which implies that if the father has experienced adverse childhood events their children will have a higher risk of experiencing emotional neglect. The results partially confirm our hypothesis.

The results suggest that the association of parental ACEs to emotional neglect experience in the offspring is small, but detectable. Although the Kruskal–Wallis test did not find a statistically significant difference between the ACEs and emotional neglect scores, the box-plot figure did show a small ascending trend. In addition, the Spearman’s correlation coefficient was minute, yet statistically significant. Only father’s ACEs and father’s education had a statistically significant correlation to child’s emotional neglect score. According to the linear regression model, the child’s emotional neglect score increased 0.3 points for each father’s ACE, which means that, as the emotional neglect score is measured in integers, it would take three ACEs to increase the child’s emotional neglect score by one point. Also, as the scale for the emotional neglect scores was 5–25, this would still be a very small increment. Nevertheless, in the light of these results parental traumas do have some effect on the children.

To the best of our knowledge, this is the first investigation into the association between parents’ ACEs and emotional neglect experienced by their children. Although it was focused specifically on ACEs and emotional neglect, its results are consistent with others obtained in slightly different settings. Christie et al. [14] and van Ee et al. [15] found in their review articles that PTSD can affect parenting behavior, which could lead to emotional neglect. Their material, however, represented extreme cases of parents with ACEs, as PTSD is a consequence of an extremely adverse (childhood) event.

One explanatory factor behind the weak correlation between parents’ ACEs and their children’s experience of emotional neglect might be resilience. This is a multidimensional concept which basically refers to a capability for positive adaptation to challenging conditions and recovery from adverse or potentially traumatic events [27]. As stated earlier, ACEs are a risk factor for mental health problems such as depression [7], but even so, not everyone who has experienced ACEs will develop depressive disorders, and this might be because of resilience [28]. Even though a parent had experienced adverse childhood events, it might still be possible, if they are resilient, to maintain a relatively stable state of mind and thus provide a good childhood for their own children. Resilience could be one way of arresting the transfer of traumas through several generations.

The setting for our study imposed some limitations. It relies on a measure of retrospective recall, as the cohort members were answering the TADS questionnaire at the age of 26 and their parents were doing so at an even greater age. This might cause some bias in the results [29]. Another source of bias may be the current mood of the subject at the moment of recall, as it might affect how that person remembers events such as ACEs that occurred in the past [30]. In addition, self-reported data might be influenced by the mindset of the participant. ACEs, especially emotional neglect, are associated with the perception of a negative attitude in others [31], and if we perceive that other people have a negative attitude toward us, we may deprecate our relationships with them. This phenomenon may have had some effect in the present instance.

This study was based on the NFBC1986, which gives a comprehensive sample of the general population in Finland. Although the source population has many strengths, our study population consisted of only 190 participants. Thus, selection bias is possible, and the results might not be generalizable at large. In addition, the distributions of children’s emotional neglect score and parents’ ACEs were quite skewed. Both parents and children had relatively low number of ACEs, as the mean score for the parents’ ACEs was 1.81 (on a scale 0–13) and the children’s mean emotional neglect score was 8.11 (on a scale 5–25). We do not know if this is due to selection bias or is this really the average level of ACEs in the NFBC1986 children and their parents. Of course, a larger population would have minimized the selection bias and provided more reliable results. Testing our hypothesis on a population with higher scores or larger deviation would also add further to the information provided by our results. Although the present work does shed some light on the possibility of parents’ ACEs affecting their children, larger samples would be needed in future to confirm these findings.

## Data Availability

The NFBC data are available from the University of Oulu, Infrastructure for Population Studies. Permission to use the data can be applied for research purposes via the electronic material request portal. Our use of the data follows the EU general data protection regulation (679/2016) and the Finnish Data Protection Act. Use of the personal data is based on each participant’s written informed consent given for the latest follow-up study, which may lead to limitations on its use. For more information, please contact the NFBC project center (NFBCprojectcenter@oulu.fi) and visit the cohort website (www.oulu.fi/nfbc).

## Supplementary material

For supplementary material accompanying this paper visit http://doi.org/10.1192/j.eurpsy.2023.2420. click here to view supplementary material
