# Supplementary material for: Emotional neglect and parents’ adverse childhood events
Source: Eur Psychiatry. 2023 Jun 9;66(1):e47. doi: 10.1192/j.eurpsy.2023.2420 (PMC10305758; doi:10.1192/j.eurpsy.2023.2420)
Supplement: Supplementary file 1 [file epasup.zip › S0924933823024203sup001.docx]

Supplementary table 1. Demographic information and TADS-scores of the included and excluded participants.

|  | Included | Excluded |
| --- | --- | --- |
| Gender N (%) |  |  |
| Women | 108 (56.8) | 164 (60.7) |
| Men | 82 (43.2) | 106 (39.3) |
| Education N (%)* |  |  |
| Level 1 | 5 (2.6) | 12 (4.4) |
| Level 2 | 78 (41.1) | 116 (43.0) |
| Level 3 | 56 (29.5) | 86 (31.9) |
| Level 4 | 51 (26.8) | 56 (20.7) |
| Marital status N (%) |  |  |
| Single | 50 (26.3) | 59 (21.9) |
| In a relationship, cohabiting or married | 137 (72.1) | 207 (76.7) |
| Divorced or widowed | 3 (1.6) | 4 (1.5) |
| TADS-score |  |  |
| Mean | 8.11 | 9.23 |
| Min | 5 | 5 |
| Max | 18 | 22 |

* If the participant reported that their education was continuing, we recorded the qualification they would reach after graduating.
