# Supplementary material for: Emotional neglect and parents’ adverse childhood events
Source: Eur Psychiatry. 2023 Jun 9;66(1):e47. doi: 10.1192/j.eurpsy.2023.2420 (PMC10305758; doi:10.1192/j.eurpsy.2023.2420)
Supplement: Supplementary file 1 [file epasup.zip › S0924933823024203sup002.docx]

Supplementary table 2. Demographic information of the parents.

|  | Mothers | Fathers |
| --- | --- | --- |
| Age at child’s birth (in years) |  |  |
| Mean | 29.6 | 31.5 |
| Min | 18 | 20 |
| Max | 48 | 47 |
| Education N (%)* |  |  |
| Level 1 | 35 (18.2) | 52 (27.4) |
| Level 2 | 120 (63.2) | 106 (55.8) |
| Level 3 | 14 (7.4) | 11 (5.8) |
| Level 4 | 13 (6.8) | 13 (6.8) |
| Missing | 8 (4.2) | 8 (4.2) |
| Marital status N (%) |  |  |
| Single | 4 (2.1) | 3 (1.6) |
| In a relationship, cohabiting or married | 163 (85.8) | 167 (87.9) |
| Divorced or widowed | 21 (11.1) | 20 (10.5) |
| Missing | 2 (1.1) | 0 |
