# Supplementary material for: Emotional neglect and parents’ adverse childhood events
Source: Eur Psychiatry. 2023 Jun 9;66(1):e47. doi: 10.1192/j.eurpsy.2023.2420 (PMC10305758; doi:10.1192/j.eurpsy.2023.2420)
Supplement: Supplementary file 1 [file epasup.zip › S0924933823024203sup003.pdf]

Questionnaire for parents

“When you think about your growth years, i.e., before you were aged 16, ...;”

1. Did your family have long-term financial problems?
2. Did your mother or father have a serious illness or disability?
3. Was your mother or father often jobless, although they would have wanted to work?
4. Did your father have problems because of alcohol?
5. Did your mother have problems because of alcohol?
6. Did your father have a mental health problem, e.g. schizophrenia, other psychosis or pression?
7. Did your mother have a mental health problem, e.g. schizophrenia, other psychosis or depression?
8. Were there serious conflicts in your family?
9. Did your parents divorce?
10. Were you seriously or chronically ill?
11. Were you bullied at school?
12. Were you abused?
13. Did someone sexually abuse you?

This data was collected in Pirkola S, Isometsä E, Aro H, Kestilä L, Hämäläinen J, Veijola J et al. Childhood adversities as risk factors for adult mental disorders: results from the Health 2000 study. Soc Psychiatry Psychiatr Epidemiol. 2005;40(10):769-77. https://doi.org/10.1007/s00127-005-0950-x.
